# Modified techniques versus Hadfield’s procedure in patients with periductal mastitis

**DOI:** 10.1186/s12893-022-01496-0

**Published:** 2022-02-05

**Authors:** Kubilay Dalci, Serdar Gumus, Ahmet Gokhan Saritas, Mehmet Onur Gul, Ahmet Rencuzogullari, Atilgan Tolga Akcam, Abdullah Ulku, Melek Ergin, Gurhan Sakman

**Affiliations:** 1 Department of General Surgery, Cukurova University, Sarıcam, 01330 Adana, Turkey; 2 Department of Surgical Oncology, Cukurova University, Sarıcam, 01330 Adana, Turkey; 3 Department of Pathology, Cukurova University Adana, Sarıcam, 01330 Adana, Turkey

**Keywords:** Abscess, Breast, Hadfield’s procedure, Periductal mastitis, Zuska’s disease

## Abstract

**Background:**

Periductal mastitis (PM) is a rare disease characterized by chronic inflammation of the terminal mammary ducts. Complete removal of terminal lactiferous ducts with Hadfield procedure is a previously defined technique in treatment but carries various complications risks. This study aims to evaluate the effectiveness of modified techniques in the treatment of PM.

**Methods:**

Twenty women who underwent surgery due to PM between January 2012 and December 2019 were retrospectively analyzed. Types of PM were determined. All patients were operated on with three different incisions [Hadfield’s operation with periareolar incision (n:11), periareolar combined radial incision (n:7), and round block incision (n:2)].

**Results:**

The mean age was 37.5 ± 6.5 years (range: 24–49). Sixty percent of patients had type 3 PM. In Hadfield’s procedure, NAC retraction (n:2), seroma (n:1), and hematoma (n:1) were seen. In the periareolar incision combined radial incision group only one patient had complications (seroma) and none in the round block method. Follow-up was 12 ± 1.5 months and disease relapse occurred in two patients in the Hadfield group. Patients who underwent round block were more satisfied with the appearance of the nipple.

**Conclusions:**

In the treatment of PM, the main principle of surgical treatment is the excision of the affected canal with a clear margin. Apart from the classical Hadfield procedure, the round block method and periareolar combined radial incision techniques can be performed in the treatment of PM.

## Introduction

Periductal mastitis (PM) is a benign disease affecting a terminal lactiferous duct responsible for 1–2% of all symptomatic breast conditions [1]. Clinically, non-cyclic mastalgia, nipple discharge, nipple-areola complex (NAC) retraction, subareolar breast mass with or without mastitis, periareolar abscess, or often lactiferous duct’s fistula can be seen [1]. The disease was first defined by Birkett [2] as the “morbid condition of the lactiferous duct” in 1850; later, in 1923, Bloodgood [3] reported that lactiferous duct enlargement and periductal inflammation take an important place in the pathogenesis. In 1951, it was defined by Zuska et al. [4] as “mammary fistula” and was referred to as Zuska’s disease in the literature.

The disease’s pathogenesis is complex, but the major pathologic finding is squamous metaplasia that acts on epithelial cells. The disease begins with periductal inflammation that develops due to the obstruction of subareolar lactiferous ducts by keratinous plaques. This inflammation causes duct rupture and periareolar fistula development [4, 5].

In the treatment; fistulotomy, fistulectomy, abscess drainage, and antibiotic therapy are not equally effective. After incision and drainage of abscess or only antibiotic therapy, there is a high probability of recurrence [1]. Therefore, due to the high recurrence rates, the technique of terminal lactiferous duct excision was described by Hadfield’ in the 1960s. However, necrosis and loss of sensation of the areola-nipple complex, nipple retraction, and postoperative infection are potential complications of the Hadfield operation. In addition, the recurrence rate after this procedure is 11% [1]. Several modifications such as a radial incision or periareolar combined radial incision have been introduced to minimize these complications [6–9].

There are no comparative articles for different surgical techniques in the literature. In our study, we tried to compare the effectiveness of modified strategies techniques, which we performed on PM.

## Methods

Twenty females who were operated on due to PM at Çukurova University Balcalı Hospital between January 2012 and December 2019 were examined.

### Data collection

**Fig. 1:**
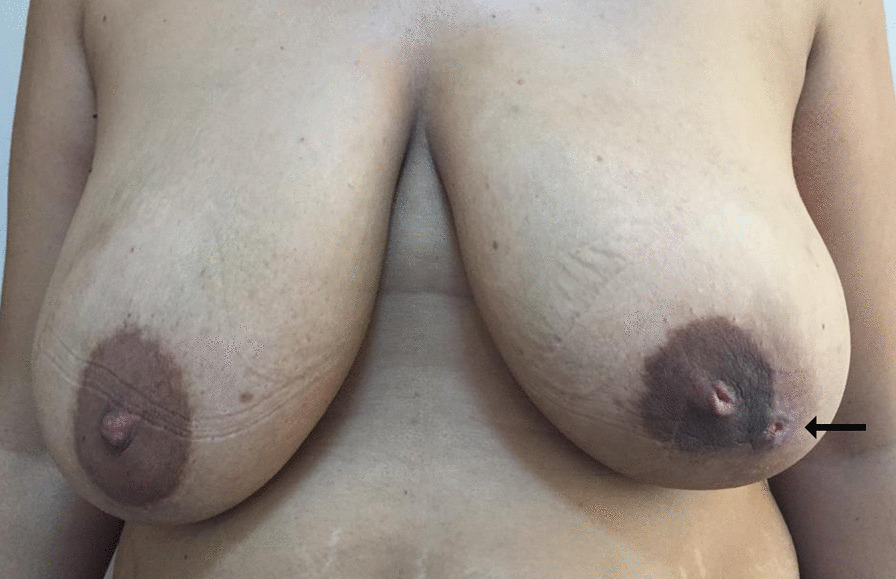
Preoperative image of PM. The fistula is located in the left breast at 5 clockwise

Detailed medical history was taken in all patients. Demographic characteristics of patients, comorbidities, previous treatment attempts and medications, etiologic risk factors (smoking history, usage of a tricyclic antidepressant, prolactinoma, and systemic lupus erythematosus), and a recurrent breast abscess with a periareolar skin opening and communication with the lactiferous duct had examined (Fig. 1). Incision type of the surgery, cosmetic results of surgery (nipple retraction after surgery), and recurrence rates were analyzed.

### Preoperative evaluation

Breast ultrasonography was performed in all patients. Patients over the age of 40 were also evaluated by digital mammography. If the breast cancer could not be distinguished, magnetic resonance imaging was also performed.

### Clinical classification

The PM classification defined by Zhang et al. [10] was used.

### Surgical technique

All surgical procedure was carried out with general anesthesia by surgeons that were specialized in the field of breast diseases. A routine prophylactic antibiotic (Cefazolin Sodium 2 g) was administered preoperatively to all patients.

**Fig. 2:**
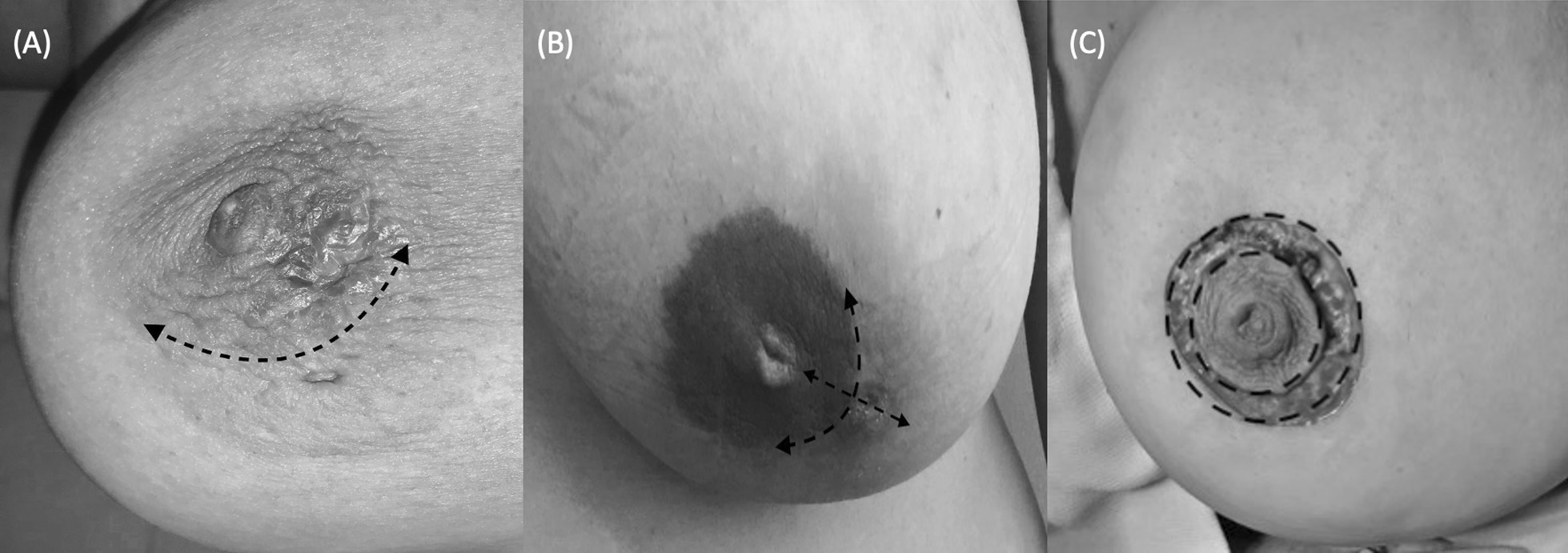
Incision types. **A** The periareolar incision for Hadfield’s operation. **B** Radial incision combined with a periareolar incision. **C** Round block incision

**Fig. 3:**
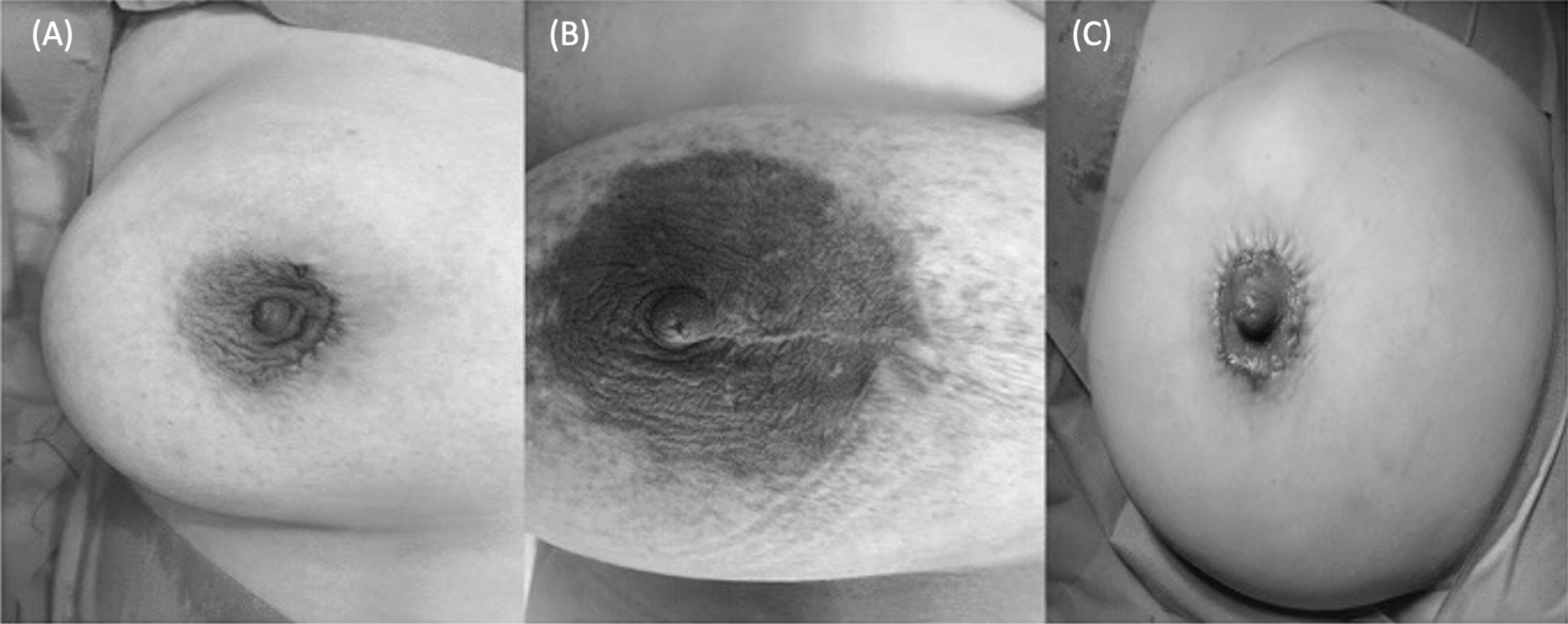
Postoperative images. **A** Periareolar incision was performed. **B** Radial incision combined with periareolar incision was performed. **C** Round block incision was performed

Three surgical procedures were performed to remove the affected duct. (i) Periareolar incision (classic Hadfield operation) [6], (ii) Periareolar incision combined with a radial incision [9], (ii) Round block incision [11] (Figs. 2, 3).

After determining the duct tract affected by palpation in all patients, the criteria we considered in the incision selection were as follows. The classic Hadfield operation with a periareolar incision was preferred if the affected duct is in the periareolar region. If the affected duct started from the NAC and lay down too far, we chose a periareolar combine radial incision. The round block incision was preferred in big-volumed breasts, and if the fistula tract is unclear or a large excision defect will occur.

The nipple is elevated off the underlying breast tissue. Then, a cone of breast tissue containing the affected ducts was excised. Post-excision volume displacement was performed with glandular flaps to avoid NAC inversion. 2/0 polyglactin was used for glandular tissue approximation, and 3/0 polyglactin was used for subcutaneous tissue closure. All skin incisions were closed subcuticular with 4/0 polyglycapron. Postoperatively, oral preparations of 500 mg of cefuroxime twice daily for 7 days were administered.

### Histopathologic evaluation

**Fig. 4:**
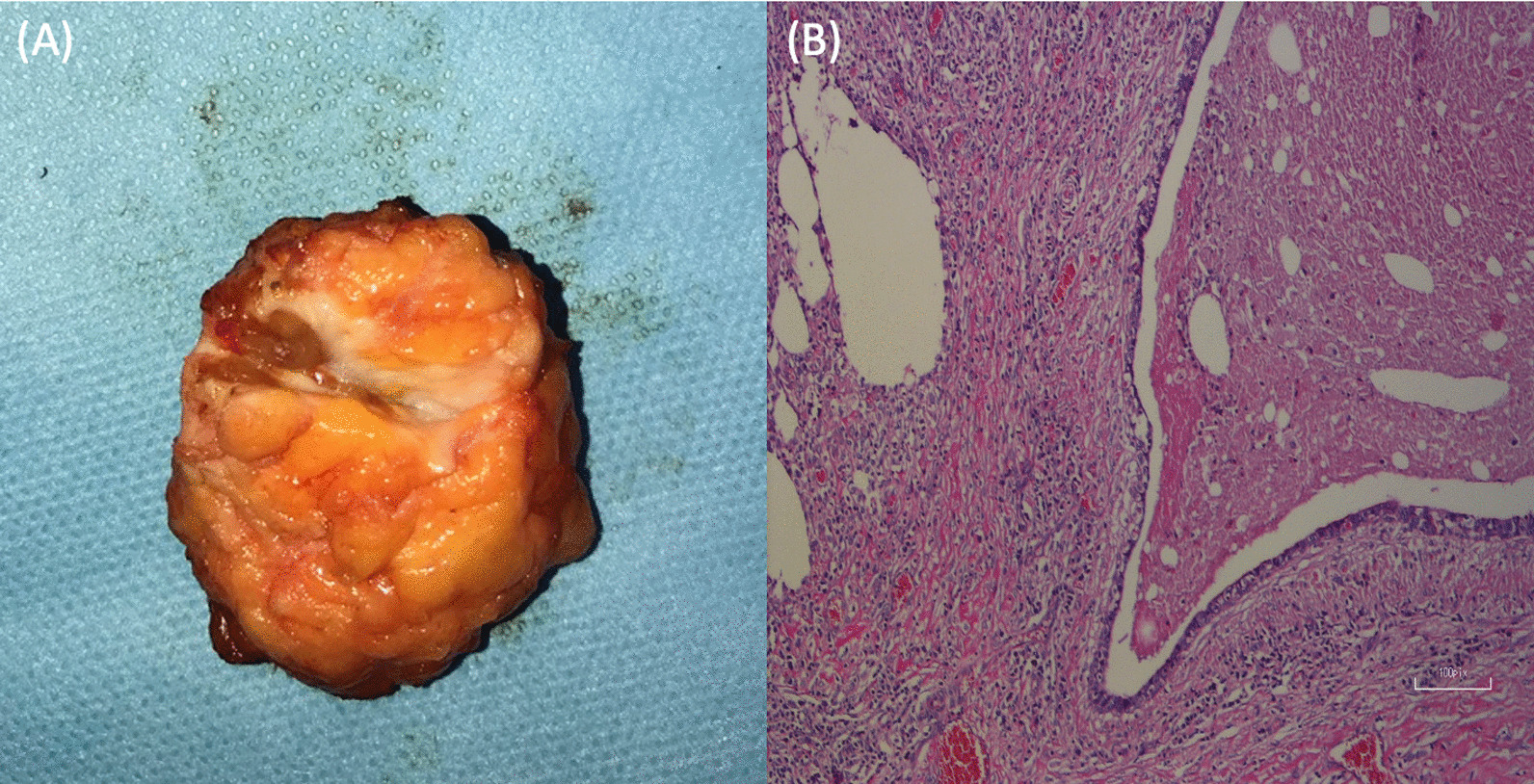
Histopathologic elevation. **A** Macroscopic imaging of the excised duct. **B** Microscopic imaging: the central duct is dilated and filled with thick secretions and there is a surrounding cuff of chronic inflammation in the periductal stroma (stained with hematoxylin and eosin)

The histopathologic features investigated were metaplastic changes in the cuboidal epithelium to the squamous epithelium, ducts obstruction by keratin plugs non-granulomatous inflammation, which was rich in plasma cells and macrophages. The definitive diagnosis of PM was confirmed histopathologically in all patients (Fig. 4).

### Follow-up

All patients were followed regularly at 1 week, 3 months, 6 months and 1 year in some, and the relapse and NAC retraction were checked in all. To evaluation of quality of life and breast and nipple cosmesis, European Organisation for Research and Treatment of Cancer QLQ-BRECON23 quality-of-life questionnaire was performed [12].

### Statistical analysis

The data were analyzed with the statistical package program SPSS v 24.0. Categorical measurements were summarized as numbers and percentages, and continuous measurements as mean deviation and minimum–maximum. The conformity of the variables to the normal distribution was examined using one of the analytical methods. Chi-square test was used to compare the groups. Paired sample t-test was used to compare the means. Results are reported as mean SD, median, number (n), and percent (%). p-value < 0.05 was considered significant.

## Results

All of the 20 patients were female. Their median age was 37.5 ± 6.5 years (ranging from 24 to 49). In fourteen cases, the disease was located in the right breast. Eight patients referred to our clinic had at least one abscess drainage before admission, and two of them were misdiagnosed as idiopathic granulomatous mastitis and received oral corticosteroid therapy. Nine patients were multipara. When possible etiological risk factors for PM are examined, sixteen patients were smokers, one patient has systemic lupus erythematosus (SLE), and two were users of tricyclic antidepressants (Table 1).

**Table 1:** Clinical and demographic features of the patients

Variable	n (%)
Lateralization	
Right	14 (70%)
Left	6 (30%)
Gravidity	
Nullipara	4 (20%)
Unipara	7 (35%)
Multipara	9 (45%)
Previous surgery	
No	12 (60%)
Abscess drainage	8 (40%)
Etiologic risk factors	
Smoking	16 (80%)
Tricyclic antidepressant	2 (10%)
Prolactinoma	0 (0%)
SLE	1 (5%)

Sixty percent of patients (n:12) had type three PM (Table 2). The surgical procedures applied to the patients are given in Table 3. As the surgical procedure, a classic Hadfield operation with periareolar incision was performed on 11 patients, periareolar incision combined radial incision was performed in 7 patients, and round block incision was performed in 2 patients.

**Table 2:** Classification of PM

Type	Findings	Descriptions	n (%)
Type I	Mass	Breast mass without abscess or fistula	4 (20%)
Type IIa	Small abscess	Breast mass with small (≤ 3 cm) abscess	1 (5%)
Type IIb	Big abscess	Breast mass with big (> 3 cm) abscess	1 (5%)
Type III	Fistula	Ductal fistula with or without breast mass	12 (60%)
Type IV	Complex or refractory	Breast mass with abscess and fistula	2 (10%)

**Table 3:** Relationship of surgical procedures with types, complications, and recurrence

	Hadfield procedure with periaerolar incision (n:11)	Periareolar combined radial incision (n:7)	Round block incision (n:2)	p
Types				
Type I	2	2	0	0.656
Type IIa	1	0	0	0.650
Type IIb	1	0	0	0.650
Type III	6	5	1	0.741
Type IV	1	0	1	0.114
Complication				
NAC retraction	2	0	0	0.403
Seroma	1	1	0	0.829
Hematoma	1	0	0	0.650
NAC necrosis	0	0	0	NA
Recurrence				
Yes	2	0	0	0.403
No	9	7	2	

Seroma was observed in only one of the patients who underwent the modified technique. In Hadfield’s procedure, NAC retraction (n:2), seroma (n:1), and hematoma (n:1) were seen. None of our patients had complications of NAC necrosis. However, there was no statistical difference in terms of complications (Table 3).

Recurrence occurred in two patients at 12 ± 1.5 months of follow-up, and both had Hadfield’s procedure (Table 3). One of these patients was treated with re-resection and the other with negative pressure wound therapy.

Table 4 presents the evaluation of patients for cosmesis and quality of life at the end of the follow-up period. Patients who underwent round block were more satisfied with their nipple appearance.

**Table 4:** EORTC QLQ-BRECON23 analysis of the patients for cosmesis

	Hadfield procedure with periaerolar incision (n:11)	Periareolar combined radial incision (n:7)	Round block incision (n:2)	p
Q60. The size of your affected breast?	2 ± 0.74	1.85 ± 0.69	3 ± 0	0.780
Q61. The shape of your affected breast?	1.91 ± 1.04	2.14 ± 0.69	3.5 ± 0.7	0.113
Q62. The appearance of the skin of your affected breast?	1.54 ± 0.68	2.14 ± 1.06	3 ± 0	0.834
Q63. The symmetry of your breasts?	2.45 ± 0.93	2.42 ± 0.97	3.5 ± 0.7	0.750
Q64. Your cleavage?	3.63 ± 0.50	3.71 ± 0.48	3 ± 0	0.235
Q65. The softness of your affected breast?	2.54 ± 0.93	2.57 ± 0.53	3 ± 0	0.860
Q66. The appearance of your affected nipple?	2.18 ± 0.87	2.85 ± 0.69	4 ± 0	0.048
Q67. The sensation in your affected nipple?	2.45 ± 1.03	2.28 ± 0.75	2.5 ± 0.7	0.340

## Discussion

Although more than 100 years have passed since the disease’s definition, there are still controversies in diagnosis, classification, and treatment. Some studies investigating the relationship between PM and smoking revealed that the amount of nicotine in the subareolar duct was higher than in plasma [10, 13, 14]. In normal breast tissues, lactiferous ducts are lined by a two-layer cuboidal epithelium, and the orifices on the nipples are lined with squamous epithelium. As a result of the cuboidal epithelium’s metaplasia to squamous epithelium, keratin plugs are formed inside the duct. The keratin plugs cause enlargement and rupture of the ducts leading to periductal inflammation and fistula development. Smoking is a known risk factor accelerating the development of such metaplasia [15]. Most of the PM cases in our study group were smokers (80%), as stated in the literature. Tricyclic antidepressant drugs inhibit dopamine secretion, thus suppressing the inhibitive effect of dopamine on prolactin secretion and might lead to hyperprolactinemia [16]. Hyperprolactinemia causes hyperplasia of the epithelium in the ducts and increases lipid- and protein-rich secretions. These secretions might lead to the terminal mammary duct’s obstruction and could develop PM [17]. Besides lactation and Tricyclic antidepressants, such autoimmune diseases as SLE, Rheumatoid Arthritis, and Sjogren’s syndrome were recorded to might cause hyperprolactinemia [18]. One patient in our series had an SLE diagnosis, and two had a history of tricyclic antidepressant use. But none of our patients had a prolactinoma.

For the first time, Zhang et al. put forward a category [19]. We grouped our patients according to their classification system, and Type 3 PM was the most common in our series. We think that this classification will be useful in determining the extent of surgery is performed.

Abscess drainage and antibiotic therapy without fistulotomy are not sufficient in PM treatment. Versluijs-Ossewaarde et al. reported a recurrence rate of 79% among patients suffering from subareolar abscess treated without excision of the terminal duct [20]. The treatment of PM includes several principles: resection of the ampulla and its abscess, a fistulectomy, reconstruction of the nipple-areola, and correction of the nipple inversion [15, 21, 22]. In 1960, Hadfield described a surgical technique for excision of the major duct system for benign disease of the breast [6]. This procedure is often used in ductal ectasia or intraductal papilloma conditions and includes a periareolar incision to remove the terminal ducts. However, the complete excision of the breast’s terminal ductal system is associated with several complications, such as nipple retraction and necrosis of NAC [7]. Taffurelli et al. presented 18 cases of PM treated with Hadfield’s procedure using a probe to find the fistula’s location. Although they reported good cosmetic results, they found that recurrence developed in 11% of cases [1]. In our series, 11 patients underwent Hadfield’s procedure, and recurrence developed in 2 of them. Moreover, complication rates were higher than other modified techniques.

Various modified surgical treatments have been reported in the literature for the PM to reduce complications and recurrence risk [23]. Menguid et al. has preferred only radial incision, which started from the middle of the NAC to encompass the diseased duct and extended laterally through the areolas and the lateral border. There was no recurrence on the side where the ductus was excised in their 24 patients. However, new fistulas developed in 4 patients in other quadrants, so they performed complete ampulla resection in a seconder surgery [8]. In another article by Komenaka et al., a combined incision of periareolar and radial incision was performed in 15 cases [9]. They found no recurrence at the same localization in the follow-up, but new fistulas developed at another quadrant in two patients. In our series, we preferred the periareolar combined radial incision in 7 patients, and we did not observe recurrence or new fistula development in any of them.

Different treatment methods in PM treatment are not limited to the choice of an incision. Some authors leave the wound to secondary healing. Beechey-Newman performed fistulectomy and saucerization with healing by secondary intention on 53 patients; however, they found an 8% recurrence rate [24]. Some surgeons prefer wide excision. But this may require flap reconstruction for closing the defect. Zhang et al. reported a recurrence rate of 4.3% in their series of 47 patients in which they close the defect after excision with dermo-glandular flap [25]. It is known that NAC retraction or necrosis is feared after such surgeries. Al Masad performed the NAC advancement as a flap into a new bed immediately above the incision across the upper half of the areola-skin junction in 33 patients. He found loss of sensation in 4%, epithelial necrosis of the upper half of the areola in 2%, and recurrence of discharge in 2% [7].

However, as seen, none of the authors stated that they used the round block method in PM’s treatment. This method is often used on oncoplastic breast‐conserving surgery for central tumors [26]. As far as we know, this study is the first article to report that the round block method can be used in the treatment of PM, and we would like to note that there was no recurrence in the two patients we treated with this method. According to our limited experience, round block and periareolar combined radial incision give better results in Types 2b, 3, and 4 PM. Because these patients have a larger abscess space or a longer fistula tract, extensive exposure is required. The classic Hadfield’s operation with a periareolar incision does not provide such extensive exposure. We think these were because we performed large excision when we use these modified procedures, and we could achieve better volume displacement.

### Limitations

Our low number of patients is the main limitation of our study due to the disease’s low incidence. The other limit is that our research was retrospective. A more extensive series or multicenter studies are required.

## Conclusion

Different surgical procedures may be preferred for the removal of the terminal milk duct in PM. The round block method and periareolar combined radial incision make a wider exposure. Therefore, both methods can be alternatives to the classical Hadfield’s procedure.

## Data Availability

The data that support the findings of this study are available from the corresponding author upon reasonable request.
